# Gene enhancer deregulation and epigenetic vulnerability

**DOI:** 10.18632/oncoscience.334

**Published:** 2016-12-21

**Authors:** Rui Lu, Gang Greg Wang

**Affiliations:** Lineberger Comprehensive Cancer Center and Department of Biochemistry and Biophysics, University of North Carolina at Chapel Hill School of Medicine, Chapel Hill, NC 27599, USA

**Keywords:** cancer epigenetics, AML, DNA methylation, DNMT3A, DOT1L

**Figure 1:**
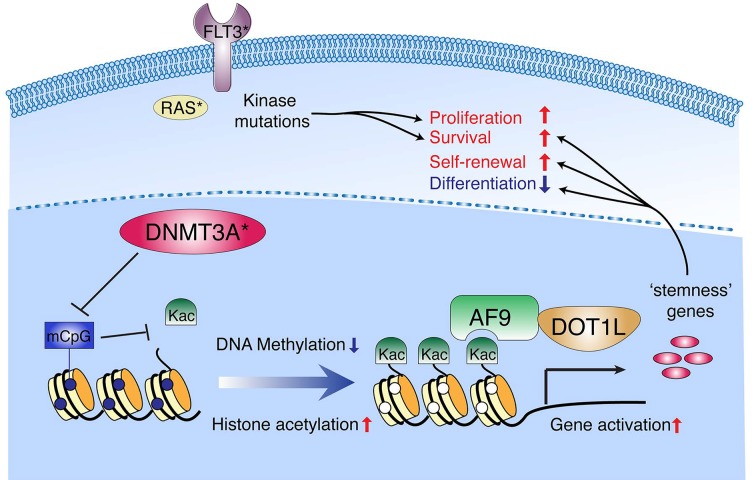
A model showing cooperativity between the mutations of DNMT3A and proliferative kinases during AML progression *, cancer-associated mutation; mCpG, methylated CpG dinucleotide; Kac, histone lysine (K) acetylation.

One major goal of cancer research is to identify tumor-specific mechanisms that sustain cell proliferation or survival and to develop the corresponding therapies that target selectively against tumor. Recent sequencing of primary tumor samples supports that aberration of chromatin modification and epigenetic states plays a central role in oncogenesis. For example, mutation of *DNA methyltransferase 3A* (DNMT3A, Figure 1) occurs in approximately 20-30% of acute myeloid leukemia (AML) and 5-15% of other hematological malignancies and disorders, making DNMT3A one of the most frequently mutated genes in blood cancer [1]; genes encoding chromatin-remodeling protein complexes are found recurrently mutated or deleted in various tumors. Thus, DNMT3A and ATP-dependent chromatin remodelers appear to function as tumor suppressors, most likely, in a context-dependent manner. However, it remains elusive how alteration of chromatin-modifying machineries contributes to tumorigenesis, and mechanism-based therapeutic approaches are to be developed.

Chromatin modifications ensure distinctive cellular identities. Past studies have shed light on several principles in chromatin modifications. One important property is reversibility. Epigenomic states are reset in response to developmental or environmental cues such as differentiation. Epigenetic changes are mediated by antagonizing enzymes that ‘write’ or ‘erase’ specific chromatin modification, exemplified by DNA methyltransferase or demethylase, and histone acetyltransferase (HAT) or deacetylase (HDAC). Second, epigenetic states can be relatively stable over cell divisions. Such ‘inheritance’ is partly owing to self-recruitment of modifying enzymes to promote self-propagation. Furthermore, different chromatin modifications that fall into the same gene-active or gene-repressive category often cooperate forming a self-reinforcement network. For example, methylated DNA is ‘read’ by MeCP2, which recruits HDACs to deacetylate histones (Figure 1). Due to the epigenetic crosstalk via antagonizing and reinforcing networks, one would predict that tumor-specific epigenetic alteration leads to subsequent aberrations in chromatin architecture, which may provide opportunity for mechanism-based therapies since chromatin-modifying enzymes are druggable.

To explore this possibility, we recently established new murine models in which a DNMT3A mutational hotspot, DNMT3AR882H, cooperates with RAS mutation to induce AML in mice [2]. We found that DNMT3AR882H confers aberrant self-renewal to hematopoietic progenitor/stem cells (HPSCs) [2]. Transcriptome analysis showed that DNMT3AR882H induces abnormally high transcription of genes crucial for HSPC self-renewal and leukemic transformation, including Meis1, Hox and Mn1 [2]. Wild-type DNMT3A suppressed expression of these ‘stemness’ genes indicating its role in safeguarding normal hematopoiesis [2]. These findings are in agreement to reports that DNMT3A mutation associates with age-related clonal hematopoiesis in normal individuals [3].

Our murine model allowed dissection of DNMT3AR882H–associated epigenetic alterations during AML progression. ChIP-sequencing of DNMT3AR882H revealed a significant overall overlap of DNMT3A binding regions with enhancers and other gene-regulatory elements [2]. To gain insight into epigenomic changes at DNMT3AR882H-targeted regions in AML cells, we performed genome-wide profiling of DNA methylation and histone modifications [2]. We found that, while wild-type DNMT3A induces CpG methylation and histone deacetylation at target sites, DNMT3AR882H resulted in focal loss of CpG methylation and increase of histone acetylation (Figure 1). Additional events that occur at DNMT3AR882H-targeted sites following its ectopic expression include the enhanced enhancer-promoter looping interaction (as detected at an Meis1 enhancer) and recruitment of HATs (such as p300) and DOT1L-AF9 complexes, which engage acetylated histones to promote transcription elongation (Figure 1). DNA hypo-methylation and/or gene-expression changes of hematopoietic ‘stemness’ genes such as Meis1, Hox and Mn1 seen in our murine AML model are reminiscent of what was observed in human AMLs with R882-mutated DNMT3A [1,2,4]. To further delineate the molecular pathways by which DNMT3AR882H contributes to oncogenesis, we carried out cause-and-effect relationship studies [2]: knockdown of Meis1 and Mn1 demonstrated AML dependency on DNMT3AR882H-activated ‘stemness’ genes; using reporter assays, we show that CpG methylation at cis-regulatory sites bound by DNMT3A indeed carries gene-repressive roles; using CRISPR/Cas9-based editing technology, we have ablated a putative Meis1 enhancer targeted by DNMT3AR882H to demonstrate a causal role for hypo-methylated enhancers in sustaining expression of ‘stemness’ genes in AML. Together, these results provided a glimpse of chain reactions initiated by mutation of a chromatin regulator in affected cancer cells.

Our work also shed light on cooperation between mutations of kinase and epigenetic factor for mediating oncogenic transformation. It is well-established that, while activating mutation of proliferative kinases such as RAS or FLT3 promotes cell hyper-proliferation, it also induces cell senescence [5], which antagonizes cancer development, and a compromised HSPC self-renewal due to over-proliferation and associated cellular stress or DNA damages [6]. Consequently, additional transformative mechanisms are required for overcoming these cancer constraints elicited by kinase mutation. Indeed, gene-expression programs activated by DNMT3AR882H such as Meis1 and Mn1 can enhance HSPC self-renewal, block differentiation, and promote tumor survival (Figure 1). Future investigation is needed to further delineate the nature of cooperativity between kinase activation and epigenetic factor mutations in cancer.

How to treat hematological cancers with loss-of-function mutation of DNMT3A? We have explored the possibility of targeting epigenetic events downstream of DNMT3AR882H with specificity-validated small-molecules. We found that DOT1L inhibitors reverse DNMT3AR882H-mediated target gene activation and selectively suppress murine AML lines with DNMT3AR882H, in comparison to those without [2]. Findings of us and others [7] support DOT1L as an epigenetic target in DNMT3A-mutated AMLs. Similarly, previous studies have shown that loss-of-function mutation of the SWI/SNF chromatin remodeling complex in tumors leads to dependency of Polycomb Repressive Complex 2 (PRC2) and that PRC2-specific inhibitors are particularly effective for treatment of these genetically defined tumors [8]. We expect that studies into other tumor subtypes carrying epigenetic factor mutations are likely to unveil similar mechanism-based interventions thus promoting a potential personalized therapeutic strategy.
